# (*E*)-3-[(3-Bromo­phen­yl)imino­meth­yl]benzene-1,2-diol: a combined X-ray and computational structural study

**DOI:** 10.1107/S1600536809035053

**Published:** 2009-09-09

**Authors:** Zeynep Keleşoğlu, Orhan Büyükgüngör, Çiğdem Albayrak, Mustafa Odabaşoğlu

**Affiliations:** a Department of Physics, Ondokuz Mayıs University, Samsun, Turkey; b Sinop Faculty of Education, Sinop University, Sinop, Turkey; c Chemical Technology Program, Denizli Higher Vocational School, Pamukkale University, Denizli, Turkey

## Abstract

The title compound, C_13_H_10_BrNO_2_, exists as an enol–imine form in the crystal and adopts an *E* configuration with respect to the C=N double bond. The mol­ecule is close to planar, with a dihedral angle of 6.88 (14)° between the aromatic rings. Intra­molecular O—H⋯N and O—H⋯O hydrogen bonds generate *S*(6) and *S*(5) ring motifs, respectively. The crystal structure is stabilized by inter­molecular O—H⋯O hydrogen-bond inter­actions, forming *R*_2_^2^(10) and *R*_2_^2^(20) chains along [100]. *ab initio* Hartree–Fock (HF), density-functional theory (DFT) and semi-empirical (AM1 and PM3) calculations and full-geometry optimizations were also performed. Although there are some discrepancies between the experimental and calculated parameters, caused presumably by the O—H⋯O hydrogen-bond inter­actions, there is an acceptable general agreement between them.

## Related literature

For general background to Schiff base compounds in coordination chemistry, see: Chen *et al.* (2008 ▶); May *et al.* (2004 ▶); Weber *et al.* (2007 ▶). For background to DFT calculations, see: Becke (1988 ▶, 1993 ▶); Lee *et al.* (1988 ▶); Schmidt & Polik *et al.* (2007 ▶); Friesner *et al.* (2005 ▶); Liu *et al.* (2004 ▶). For a related structure, see: Cao *et al.* (2009 ▶); Temel *et al.* (2007 ▶). For hydrogen-bond motifs, see: Bernstein *et al.* (1995 ▶).
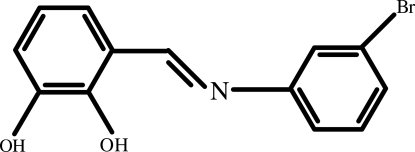


## Experimental

### 

#### Crystal data

C_13_H_10_BrNO_2_
*M* *_r_* = 292.13
Orthorhombic, 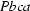

*a* = 4.7411 (2) Å
*b* = 18.9447 (6) Å
*c* = 26.1417 (10) Å
*V* = 2348.01 (15) Å^3^
*Z* = 8
Mo *K*α radiation
μ = 3.50 mm^−1^
*T* = 296 K
0.66 × 0.38 × 0.10 mm

#### Data collection

Stoe IPDS-II diffractometer
Absorption correction: integration (*X-RED32*; Stoe & Cie, 2002 ▶) *T*_min_ = 0.229, *T*_max_ = 0.735
7185 measured reflections
2212 independent reflections
1764 reflections with *I* > 2σ(*I*)
*R*_int_ = 0.040

#### Refinement

*R*[*F* ^2^ > 2σ(*F* ^2^)] = 0.033
*wR*(*F* ^2^) = 0.078
*S* = 1.05
2212 reflections
162 parameters
H atoms treated by a mixture of independent and constrained refinement
Δρ_max_ = 0.33 e Å^−3^
Δρ_min_ = −0.42 e Å^−3^

### 

Data collection: *X-AREA* (Stoe & Cie, 2002 ▶); cell refinement: *X-AREA*; data reduction: *X-RED32* (Stoe & Cie, 2002 ▶); program(s) used to solve structure: *SHELXS97* (Sheldrick, 2008 ▶); program(s) used to refine structure: *SHELXL97* (Sheldrick, 2008 ▶); molecular graphics: *ORTEP-3 for Windows* (Farrugia, 1997 ▶); software used to prepare material for publication: *WinGX* (Farrugia, 1999 ▶) and *GAUSSIAN* (Frisch *et al*., 2004 ▶).

## Supplementary Material

Crystal structure: contains datablocks I, global. DOI: 10.1107/S1600536809035053/si2198sup1.cif

Structure factors: contains datablocks I. DOI: 10.1107/S1600536809035053/si2198Isup2.hkl

Additional supplementary materials:  crystallographic information; 3D view; checkCIF report

## Figures and Tables

**Table 1:** Hydrogen-bond geometry (Å, °)

*D*—H⋯*A*	*D*—H	H⋯*A*	*D*⋯*A*	*D*—H⋯*A*
O1—H1⋯N1	0.87 (4)	1.81 (4)	2.606 (3)	151 (3)
O2—H2⋯O1	0.78 (4)	2.31 (5)	2.718 (3)	113 (4)
O2—H2⋯O2^i^	0.78 (4)	2.48 (5)	3.124 (3)	141 (5)
O2—H2⋯O1^ii^	0.78 (4)	2.46 (4)	2.986 (3)	126 (4)

Symmetry codes: (i) 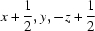; (ii) 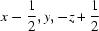.

**Table:** 

Parameters	X-ray	AM1	PM3	HF*	DFT/B3LYP*
C1—C7	1.447 (4)	1.4659	1.4592	1.4655	1.4472
C8—N1	1.416 (3)	1.4103	1.431	1.4082	1.4071
C7—N1	1.278 (3)	1.2923	1.3028	1.2626	1.2947
C2—O1	1.355 (3)	1.3711	1.3612	1.3414	1.35
C3—O2	1.358 (3)	1.3749	1.3695	1.3472	1.3601
C10—Br1	1.900 (2)	1.8743	1.8676	1.899	1.9138

**Table:** 

O1—C2—C1	122.8 (2)	126.384	124.0177	124.2818	123.5134
N1—C7—C1	122.1 (2)	123.752	119.6344	123.297	121.9975
O2—C3—C4	119.9 (2)	117.2553	115.9182	119.9887	120.7548
O1—C2—C3	117.5 (2)	113.7932	116.4985	115.8053	116.4318
O2—C3—C2	120.5 (2)	122.181	123.9237	119.978	119.4331
C7—N1—C8	121.6 (2)	121.8246	122.1744	120.3634	121.3341

**Table:** 

C12—C13—H13	119.6	119.7856	119.8376	120.8687	121.0058
C8—C13—H13	119.6	120.1274	120.1469	119.0149	118.759
C1—C7—N1—C8	179.7 (2)	−179.2308	179.9974	−178.6515	−177.5099
C9—C8—N1—C7	7.9 (4)	34.1092	0.0009	44.5418	35.1166
C2—C1—C7—N1	−1.6 (4)	2.6542	0.0087	0.8066	0.3196
N1—C8—C9—C10	−179.9 (2)	−177.3895	179.9976	179.3862	179.4699
C8—C9—C10—Br1	−179.41 (19)	−179.8397	−180.0011	−179.9136	−179.7804

*6-31G(d,p).

**Fig. 1.**
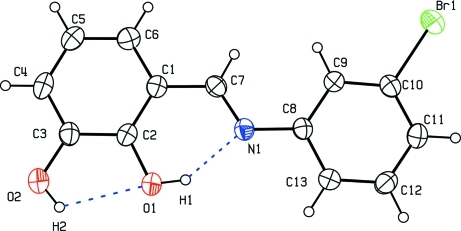
An ORTEP view of (I), with the atom-numbering scheme and 30% probability displacement ellipsoids. Dashed lines indicate H-bonds.

**Fig. 2.**
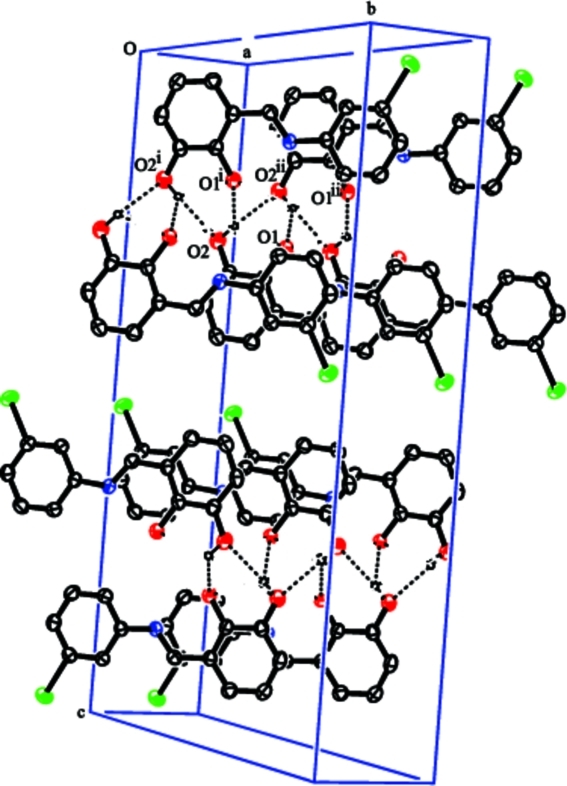
A packing diagram for (I), showing the O—H···O hydrogen bonds, forming R22(10) and R22(20) chains at [100]. [Symmetry codes; (i): x + 1/2, y, -z + 1/2; (ii): x - 1/2, y, -z + 1/2]. H atoms not involved in hydrogen bonding (dashed lines) have been omitted for clarity.

## supplementary crystallographic information

### Comment

Schiff base compounds have received considerable attention for many years, primarily due to their importance in the development of coordination chemistry related to magnetism (Weber *et al.*, 2007), catalysis (Chen *et al.*, 2008) and biological process (May *et al.*, 2004). In general, *O*-hydroxy Schiff bases exhibit two possible tautomeric forms, the enol-imine and keto-amine forms. Depending on the tautomers, two types of intra-molecular hydrogen bonds are possible: O—H···N in enol-imine and N—H···O in keto-amine form.

The molecule adopts an E configuration with respect to the C7=N1 double bond, with a C1—C7=N1—C8 torsion angle of 179.7 (2)° and a C7=N1—C8 angle of 121.6 (2)°. Similar results were observed for (*E*)-3-[(2-Bromophenyl) iminomethyl]-benzene-1,2-diol [178.4 (2) and 123.4 (2)°; Temel *et al.*,2007]. The C7=N1 bond length is 1.278 (3) Å, and agree with the corresponding distance in (*E*)-)-3-Bromo-*N*'-(4-hydroxy-3-nitrobenzylidene) benzohydrazide[1.276 (4) Å; Cao *et al.*,2009]. Intramolecular O—H···N and O—H···O hydrogen bonds generate S(6) and S(5) ring motifs (Bernstein *et al.*, 1995) (Fig. 1). The intermolecular O2—H2..O2 and O2—H2..O1 hydrogen bonds in the molecule at (*x* + 1/2, *y*, -*z* + 1/2)and (*x* - 1/2, *y*, -*z* + 1/2), forming *R*_2_^2^(10) and *R*_2_^2^(20) chains at [100] direction.(Table 1, Fig.2). The dihedral angle between benzene rings A(C1—C6) and B(C8—C13) is 6.88 (15)°. The planar S(6) ring C(O1/H1/N1/C1/C2/C7) is oriented with respect to rings A and B at dihedral angles of 0.16 (44)° and 6.88 (41)°, respectively. These dihedral angles show that the molecule of (I) is almost planar. It is known that Schiff bases may exhibit thermochromism or photochromism, depending on the planarity or non-planarity of the molecule, respectively. Since the title molecule is planar, one can expect thermochromic properties in title compound.

Ab-*initio* Hartree-Fock (HF), density-functional theory (DFT) (Schmidt & Polik, 2007) and semi-empirical (AM1 and PM3) calculations and full-geometry optimizations were performed by means of *GAUSSIAN* 03 W package (Frisch *et al.*, 2004). The selected bond lengths and angles together with the torsion angles are compared with the obtained ones from semi-empirical, *ab*-*initio* HF and DFT/B3-LYP (Becke 3 parameter Lee-Yang-Parr) (Becke, 1988, 1993; Lee *et al.*,  1988) (Table 2). We observe an acceptable general agreement between them. Although the DFT molecular orbital theory was considered as the most accurate method for geometry optimization for free and complex ligands (Friesner, 2005; Liu *et al.*, 2004), the HF method led to better results in regard to the bond lengths and angles.

### Experimental

For the preparation of compound (I) the mixture of 2,3-dihydroxybenzaldehyde (0.5 g, 3.6 mmol) in ethanol (20 ml) and 3-bromoaniline (0.62 g, 3.6 mmol) in ethanol (20 ml) was stirred for 1 h under reflux. The crystals suitable for X-ray analysis were obtained from ethanol by slow evaporation (yield; %88, m.p.; 402–403 K).

### Refinement

Due to their taking part in H-bonding interactions, the hydroxyl H atoms were preferred to locate in difference Fourier map and refined freely with *U*_iso_(H) = 1.5 *U*_eq_(O). All other H-atoms were refined using a riding model with d(C—H)= 0.93 Å and *U*_iso_(H)= 1.2 *U*_eq_(C).

### Crystal data

**Table:**

C_13_H_10_BrNO_2_	*F*(000) = 1168
*M**_r_* = 292.13	*D*_x_ = 1.653 Mg m^−^^3^
Orthorhombic, *P**b**c**a*	Mo *K*α radiation, λ = 0.71073 Å
Hall symbol: -P 2ac 2ab	Cell parameters from 7185 reflections
*a* = 4.7411 (2) Å	θ = 1.3–26.2°
*b* = 18.9447 (6) Å	µ = 3.50 mm^−^^1^
*c* = 26.1417 (10) Å	*T* = 296 K
*V* = 2348.01 (15) Å^3^	Plate, red
*Z* = 8	0.66 × 0.38 × 0.10 mm

### Data collection

**Table:**

Stoe IPDS-II diffractometer	2212 independent reflections
Radiation source: fine-focus sealed tube	1764 reflections with *I* > 2σ(*I*)
graphite	*R*_int_ = 0.040
Detector resolution: 6.67 pixels mm^-1^	θ_max_ = 25.6°, θ_min_ = 1.6°
rotation method scans	*h* = −5→5
Absorption correction: integration (*X-RED32*; Stoe & Cie, 2002)	*k* = −22→22
*T*_min_ = 0.229, *T*_max_ = 0.735	*l* = −31→31
7185 measured reflections	

### Refinement

**Table:**

Refinement on *F*^2^	Secondary atom site location: difference Fourier map
Least-squares matrix: full	Hydrogen site location: inferred from neighbouring sites
*R*[*F*^2^ > 2σ(*F*^2^)] = 0.033	H atoms treated by a mixture of independent and constrained refinement
*wR*(*F*^2^) = 0.078	*w* = 1/[σ^2^(*F*_o_^2^) + (0.0356*P*)^2^ + 0.8167*P*] where *P* = (*F*_o_^2^ + 2*F*_c_^2^)/3
*S* = 1.05	(Δ/σ)_max_ = 0.002
2212 reflections	Δρ_max_ = 0.33 e Å^−^^3^
162 parameters	Δρ_min_ = −0.42 e Å^−^^3^
0 restraints	Extinction correction: *SHELXL97* (Sheldrick, 2008)
Primary atom site location: structure-invariant direct methods	Extinction coefficient: 0

### Special details

**Table:**

Geometry. All esds (except the esd in the dihedral angle between two l.s. planes) are estimated using the full covariance matrix. The cell esds are taken into account individually in the estimation of esds in distances, angles and torsion angles; correlations between esds in cell parameters are only used when they are defined by crystal symmetry. An approximate (isotropic) treatment of cell esds is used for estimating esds involving l.s. planes.
Refinement. Refinement of F^2^ against ALL reflections. The weighted R-factor wR and goodness of fit S are based on F^2^, conventional R-factors R are based on F, with F set to zero for negative F^2^. The threshold expression of F^2^ > 2sigma(F^2^) is used only for calculating R-factors(gt) etc. and is not relevant to the choice of reflections for refinement. R-factors based on F^2^ are statistically about twice as large as those based on F, and R- factors based on ALL data will be even larger.

### Fractional atomic coordinates and isotropic or equivalent isotropic displacement parameters (Å^2^)

**Table:**

	*x*	*y*	*z*	*U*_iso_*/*U*_eq_	
C1	0.2287 (5)	0.59650 (12)	0.38493 (9)	0.0430 (5)	
C2	0.1687 (5)	0.56587 (13)	0.33762 (9)	0.0441 (5)	
C3	−0.0340 (6)	0.51215 (13)	0.33440 (10)	0.0480 (6)	
C4	−0.1727 (6)	0.49065 (14)	0.37771 (11)	0.0543 (7)	
H4	−0.3082	0.4553	0.3753	0.065*	
C5	−0.1148 (6)	0.52049 (15)	0.42490 (11)	0.0556 (7)	
H5	−0.2106	0.5052	0.4539	0.067*	
C6	0.0848 (6)	0.57283 (14)	0.42865 (10)	0.0524 (6)	
H6	0.1248	0.5927	0.4603	0.063*	
C7	0.4352 (5)	0.65240 (13)	0.38933 (9)	0.0451 (6)	
H7	0.4674	0.6726	0.4212	0.054*	
C8	0.7753 (5)	0.72987 (12)	0.35557 (9)	0.0409 (5)	
C9	0.8643 (5)	0.75859 (13)	0.40177 (9)	0.0461 (6)	
H9	0.7918	0.7419	0.4325	0.055*	
C10	1.0603 (6)	0.81192 (13)	0.40147 (9)	0.0475 (6)	
C11	1.1712 (6)	0.83889 (14)	0.35705 (10)	0.0520 (6)	
H11	1.3031	0.8752	0.3578	0.062*	
C12	1.0810 (6)	0.81048 (15)	0.31139 (10)	0.0572 (7)	
H12	1.1510	0.8283	0.2808	0.069*	
C13	0.8888 (6)	0.75614 (15)	0.31054 (10)	0.0513 (6)	
H13	0.8342	0.7368	0.2794	0.062*	
N1	0.5747 (4)	0.67489 (11)	0.35080 (8)	0.0442 (5)	
O1	0.2990 (5)	0.58565 (11)	0.29377 (7)	0.0583 (5)	
O2	−0.0900 (5)	0.48050 (12)	0.28892 (8)	0.0684 (6)	
Br1	1.18395 (9)	0.85040 (2)	0.464746 (12)	0.08086 (16)	
H1	0.415 (8)	0.619 (2)	0.3026 (13)	0.088 (12)*	
H2	−0.007 (10)	0.499 (2)	0.2666 (14)	0.113 (16)*	

### Atomic displacement parameters (Å^2^)

**Table:**

	*U*^11^	*U*^22^	*U*^33^	*U*^12^	*U*^13^	*U*^23^
C1	0.0352 (13)	0.0422 (12)	0.0517 (13)	0.0035 (10)	−0.0009 (10)	0.0026 (10)
C2	0.0396 (13)	0.0415 (13)	0.0513 (13)	−0.0004 (11)	0.0019 (11)	0.0055 (11)
C3	0.0454 (14)	0.0416 (13)	0.0570 (14)	−0.0023 (11)	−0.0068 (12)	0.0033 (11)
C4	0.0431 (15)	0.0433 (14)	0.0764 (18)	−0.0048 (12)	0.0014 (13)	0.0124 (13)
C5	0.0503 (16)	0.0549 (16)	0.0616 (16)	−0.0008 (13)	0.0088 (13)	0.0107 (13)
C6	0.0495 (15)	0.0553 (15)	0.0525 (14)	0.0032 (13)	0.0019 (12)	0.0019 (12)
C7	0.0432 (13)	0.0457 (13)	0.0465 (12)	0.0027 (11)	−0.0039 (11)	−0.0036 (10)
C8	0.0359 (12)	0.0393 (12)	0.0476 (12)	0.0023 (10)	−0.0044 (10)	0.0006 (9)
C9	0.0475 (15)	0.0472 (14)	0.0436 (12)	−0.0054 (12)	−0.0012 (11)	0.0028 (10)
C10	0.0485 (15)	0.0441 (14)	0.0498 (13)	−0.0009 (12)	−0.0058 (12)	−0.0038 (11)
C11	0.0490 (15)	0.0440 (14)	0.0630 (15)	−0.0050 (12)	0.0027 (13)	−0.0010 (11)
C12	0.0615 (18)	0.0578 (16)	0.0524 (14)	−0.0089 (14)	0.0103 (13)	0.0035 (12)
C13	0.0551 (16)	0.0548 (16)	0.0441 (13)	−0.0062 (13)	−0.0012 (12)	−0.0012 (11)
N1	0.0396 (11)	0.0440 (11)	0.0490 (11)	−0.0024 (9)	−0.0028 (9)	0.0001 (9)
O1	0.0626 (12)	0.0612 (12)	0.0512 (10)	−0.0231 (10)	0.0022 (9)	−0.0014 (9)
O2	0.0769 (15)	0.0665 (14)	0.0618 (12)	−0.0291 (12)	−0.0049 (11)	−0.0011 (10)
Br1	0.1009 (3)	0.0846 (2)	0.05706 (19)	−0.0355 (2)	−0.01118 (17)	−0.01177 (16)

### Geometric parameters (Å, °)

**Table:**

C1—C2	1.395 (3)	C8—C13	1.387 (3)
C1—C6	1.405 (4)	C8—C9	1.390 (3)
C1—C7	1.447 (4)	C8—N1	1.416 (3)
C2—O1	1.355 (3)	C9—C10	1.373 (4)
C2—C3	1.402 (3)	C9—H9	0.9300
C3—O2	1.358 (3)	C10—C11	1.373 (4)
C3—C4	1.371 (4)	C10—Br1	1.900 (2)
C4—C5	1.384 (4)	C11—C12	1.377 (4)
C4—H4	0.9300	C11—H11	0.9300
C5—C6	1.374 (4)	C12—C13	1.375 (4)
C5—H5	0.9300	C12—H12	0.9300
C6—H6	0.9300	C13—H13	0.9300
C7—N1	1.278 (3)	O1—H1	0.87 (4)
C7—H7	0.9300	O2—H2	0.78 (4)
			
C2—C1—C6	119.3 (2)	C13—C8—C9	118.6 (2)
C2—C1—C7	120.9 (2)	C13—C8—N1	116.7 (2)
C6—C1—C7	119.8 (2)	C9—C8—N1	124.6 (2)
O1—C2—C1	122.8 (2)	C10—C9—C8	119.2 (2)
O1—C2—C3	117.5 (2)	C10—C9—H9	120.4
C1—C2—C3	119.7 (2)	C8—C9—H9	120.4
O2—C3—C4	119.9 (2)	C9—C10—C11	122.5 (2)
O2—C3—C2	120.5 (2)	C9—C10—Br1	119.09 (19)
C4—C3—C2	119.7 (2)	C11—C10—Br1	118.4 (2)
C3—C4—C5	121.3 (3)	C10—C11—C12	117.9 (2)
C3—C4—H4	119.4	C10—C11—H11	121.0
C5—C4—H4	119.3	C12—C11—H11	121.0
C6—C5—C4	119.6 (3)	C13—C12—C11	120.8 (3)
C6—C5—H5	120.2	C13—C12—H12	119.6
C4—C5—H5	120.2	C11—C12—H12	119.6
C5—C6—C1	120.5 (3)	C12—C13—C8	120.8 (2)
C5—C6—H6	119.8	C12—C13—H13	119.6
C1—C6—H6	119.8	C8—C13—H13	119.6
N1—C7—C1	122.1 (2)	C7—N1—C8	121.6 (2)
N1—C7—H7	118.9	C2—O1—H1	105 (2)
C1—C7—H7	118.9	C3—O2—H2	111 (3)
			
C6—C1—C2—O1	−179.8 (2)	C6—C1—C7—N1	178.8 (2)
C7—C1—C2—O1	0.6 (4)	C13—C8—C9—C10	0.1 (4)
C6—C1—C2—C3	0.1 (4)	N1—C8—C9—C10	−179.9 (2)
C7—C1—C2—C3	−179.5 (2)	C8—C9—C10—C11	0.7 (4)
O1—C2—C3—O2	1.6 (4)	C8—C9—C10—Br1	−179.41 (19)
C1—C2—C3—O2	−178.3 (2)	C9—C10—C11—C12	−0.4 (4)
O1—C2—C3—C4	−179.6 (2)	Br1—C10—C11—C12	179.8 (2)
C1—C2—C3—C4	0.5 (4)	C10—C11—C12—C13	−0.9 (4)
O2—C3—C4—C5	178.2 (3)	C11—C12—C13—C8	1.7 (4)
C2—C3—C4—C5	−0.6 (4)	C9—C8—C13—C12	−1.3 (4)
C3—C4—C5—C6	0.2 (4)	N1—C8—C13—C12	178.7 (2)
C4—C5—C6—C1	0.4 (4)	C1—C7—N1—C8	179.7 (2)
C2—C1—C6—C5	−0.5 (4)	C13—C8—N1—C7	−172.2 (2)
C7—C1—C6—C5	179.0 (2)	C9—C8—N1—C7	7.9 (4)
C2—C1—C7—N1	−1.6 (4)		

### Hydrogen-bond geometry (Å, °)

**Table:**

*D*—H···*A*	*D*—H	H···*A*	*D*···*A*	*D*—H···*A*
O1—H1···N1	0.87 (4)	1.81 (4)	2.606 (3)	151 (3)
O2—H2···O1	0.78 (4)	2.31 (5)	2.718 (3)	113 (4)
O2—H2···O2^i^	0.78 (4)	2.48 (5)	3.124 (3)	141 (5)
O2—H2···O1^ii^	0.78 (4)	2.46 (4)	2.986 (3)	126 (4)

Symmetry codes: (i) *x*+1/2, *y*, −*z*+1/2; (ii) *x*−1/2, *y*, −*z*+1/2.

### Table 2 Selected geometric parameters (Å, °) from the X-ray structure and calculated by AM1, PM3, HF and DFT methods

**Table:**

Parameters	X-ray	AM1	PM3	HF*	DFT/B3LYP*
C1—C7	1.447 (4)	1.4659	1.4592	1.4655	1.4472
C8—N1	1.416 (3)	1.4103	1.431	1.4082	1.4071
C7—N1	1.278 (3)	1.2923	1.3028	1.2626	1.2947
C2—O1	1.355 (3)	1.3711	1.3612	1.3414	1.35
C3—O2	1.358 (3)	1.3749	1.3695	1.3472	1.3601
C10—Br1	1.900 (2)	1.8743	1.8676	1.899	1.9138
O1—C2—C1	122.8 (2)	126.384	124.0177	124.2818	123.5134
N1—C7—C1	122.1 (2)	123.752	119.6344	123.297	121.9975
O2—C3—C4	119.9 (2)	117.2553	115.9182	119.9887	120.7548
O1—C2—C3	117.5 (2)	113.7932	116.4985	115.8053	116.4318
O2—C3—C2	120.5 (2)	122.181	123.9237	119.978	119.4331
C7—N1—C8	121.6 (2)	121.8246	122.1744	120.3634	121.3341
C12—C13—H13	119.6	119.7856	119.8376	120.8687	121.0058
C8—C13—H13	119.6	120.1274	120.1469	119.0149	118.759
C1—C7—N1—C8	179.7 (2)	-179.2308	179.9974	-178.6515	-177.5099
C9—C8—N1—C7	7.9 (4)	34.1092	0.0009	44.5418	35.1166
C2—C1—C7—N1	-1.6 (4)	2.6542	0.0087	0.8066	0.3196
N1—C8—C9—C10	-179.9 (2)	-177.3895	179.9976	179.3862	179.4699
C8—C9—C10—Br1	-179.41 (19)	-179.8397	-180.0011	-179.9136	-179.7804

*6-31G(d,p).

## References

[bb1] Becke, A. D. (1988). *Phys. Rev. A*, **38**, 3098–100. 10.1103/physreva.38.3098 9900728

[bb2] Becke, A. D. (1993). *J. Chem. Phys.* **98**, 5648–5652.

[bb3] Bernstein, J., Davis, R. E., Shimoni, L. & Chang, N.-L. (1995). *Angew. Chem. Int. Ed. Engl.* **34**, 1555–1573.

[bb4] Cao, G.-B. & Wang, X.-Y. (2009). *Acta Cryst.* E**65**, o1725. 10.1107/S1600536809024131 PMC2977221 21583442

[bb5] Chen, Z. H., Morimoto, H., Matsunaga, S. & Shibasaki, M. (2008). *J. Am. Chem. Soc.* **130**, 2170–2171. 10.1021/ja710398q 18225906

[bb6] Farrugia, L. J. (1997). *J. Appl. Cryst.* **30**, 565.

[bb7] Farrugia, L. J. (1999). *J. Appl. Cryst.* **32**, 837–838.

[bb8] Friesner, R. A. (2005). *Proc. Natl Acad. Sci. USA*, **102**, 6648–6653. 10.1073/pnas.0408036102 PMC1100737 15870212

[bb9] Frisch, M. J., *et al.* (2004). *GAUSSIAN03* Gaussian Inc., Wallingford, CT 06492, USA.

[bb10] Lee, C., Yang, W. & Parr, R. G. (1988). *Phys. Rev. B*, **37**, 785–789. 10.1103/physrevb.37.785 9944570

[bb11] Liu, H., Bandeira, N. A. G., Calhorda, M. J., Drew, M. G. B., Felix, V., Novosad, J., De Biani, F. F. & Zanello, P. (2004). *J. Organomet. Chem.* **689**, 2808–2819.

[bb12] May, J. P., Ting, R., Lermer, L., Thomas, J. M., Roupioz, Y. & Perrin, D. M. (2004). *J. Am. Chem. Soc.* **126**, 4145–4156. 10.1021/ja037625s 15053604

[bb13] Schmidt, J. R. & Polik, W. F. (2007). *WebMO Pro* WebMO, LLC: Holland, MI, USA; available from http://www.webmo.net.

[bb14] Sheldrick, G. M. (2008). *Acta Cryst.* A**64**, 112–122. 10.1107/S0108767307043930 18156677

[bb15] Stoe & Cie (2002). *X-AREA* and *X-RED32* Stoe & Cie, Darmstadt, Germany.

[bb16] Temel, E., Albayrak, Ç., Odabaşoğlu, M. & Büyükgüngör, O. (2007). *Acta Cryst.* E**63**, o1319–o1320.

[bb17] Weber, B., Tandon, R. & Himsl, D. (2007). *Z. Anorg. Allg. Chem.* **633**, 1159–1162.

